# Transcranial Alternating Current Stimulation (tACS) Mechanisms and Protocols

**DOI:** 10.3389/fncel.2017.00214

**Published:** 2017-09-01

**Authors:** Amir V. Tavakoli, Kyongsik Yun

**Affiliations:** ^1^Division of Biology and Biological Engineering, California Institute of Technology Pasadena, CA, United States; ^2^Department of Psychology, University of California, Los Angeles Los Angeles, CA, United States; ^3^Computation and Neural Systems, California Institute of Technology Pasadena, CA, United States; ^4^Bio-Inspired Technologies and Systems, Jet Propulsion Laboratory, California Institute of Technology Pasadena, CA, United States

**Keywords:** transcranial alternating current stimulation (tACS), transcranial electrical stimulation (tES), neuromodulation, noninvasive brain stimulation (NIBS), neuroplasticity, cognitive performance, neural entrainment, non-invasive transcranial brain stimulation (NTBS)

## Abstract

Perception, cognition and consciousness can be modulated as a function of oscillating neural activity, while ongoing neuronal dynamics are influenced by synaptic activity and membrane potential. Consequently, transcranial alternating current stimulation (tACS) may be used for neurological intervention. The advantageous features of tACS include the biphasic and sinusoidal tACS currents, the ability to entrain large neuronal populations, and subtle control over somatic effects. Through neuromodulation of phasic, neural activity, tACS is a powerful tool to investigate the neural correlates of cognition. The rapid development in this area requires clarity about best practices. Here we briefly introduce tACS and review the most compelling findings in the literature to provide a starting point for using tACS. We suggest that tACS protocols be based on functional brain mechanisms and appropriate control experiments, including active sham and condition blinding.

## Introduction

Technological and ethical constraints have forced the study of human cognition to rely on non-invasive electrophysiology and neuroimaging techniques to reveal the neural correlates of perception, cognition and behavioral functions. Through electrochemistry and neuronal cytology, we have learned that neurophysiological dynamics determine neural function (Bullmore and Sporns, 2009). Transcranial alternating current stimulation (tACS) has emerged with particular advantages, given its ability to probe the causal neurophysiology underlying function. As the evolution of transcranial electric current stimulation (tES), tACS is a means of non-invasive brain stimulation (NIBS) that helps us strengthen neuroscientific inferences (Riecke et al., 2015a; Santarnecchi et al., 2017).

**Figure 1:**
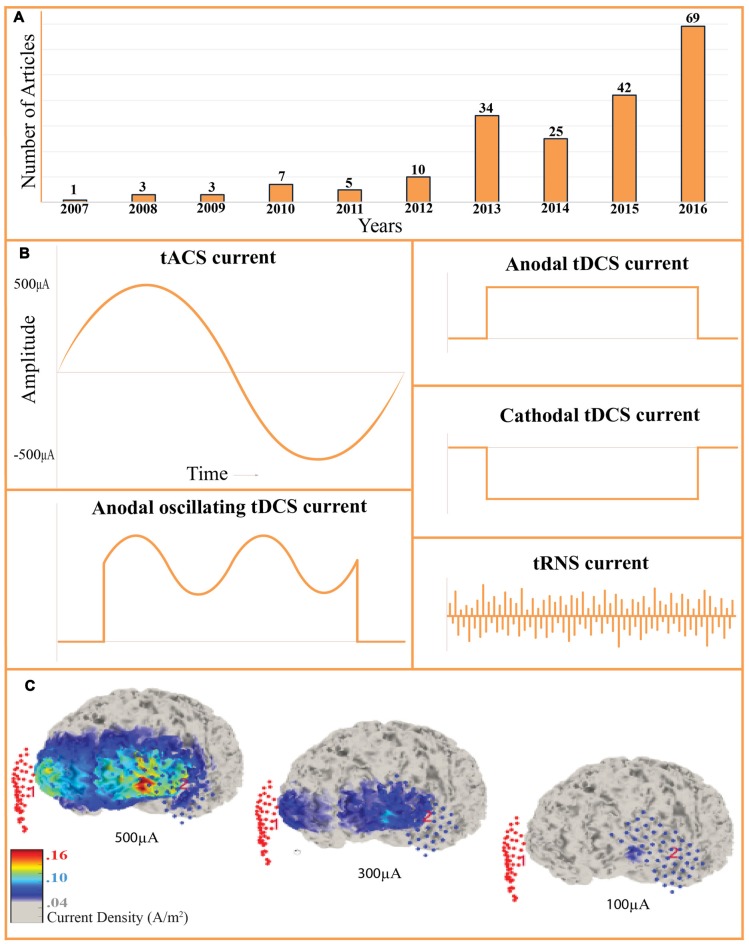
Recent growth and the transcranial alternating current stimulation (tACS) current profile. **(A)** Number of tACS studies in the last 10 years. PubMed listed articles that used the term “tACS” in the title or in the abstract were counted. The technique has been increasingly applied in the recent years and we may predict the exponential increase in the number of studies in the upcoming years. **(B)** Transcranial current stimulation protocols. tACS, transcranial alternating current stimulation; tDCS, transcranial direct current stimulation; tRNS, transcranial random noise stimulation; otDCS, oscillating tDCS. **(C)** Computational modeling of cortical current density while stimulating with tACS. 5 cm × 5 cm electrodes were placed on the F3 and F4. Three brains represent 500 μA, 300 μA and 100 μA stimulation. Visualized with COMETS2 toolbox for MATLAB, the size of the affected cortical regions and the current density both increase as the stimulation is increased. Stimulation intensity and electrode size should be carefully determined based on the size of the target region (Lee et al., 2017).

**Figure 2:**
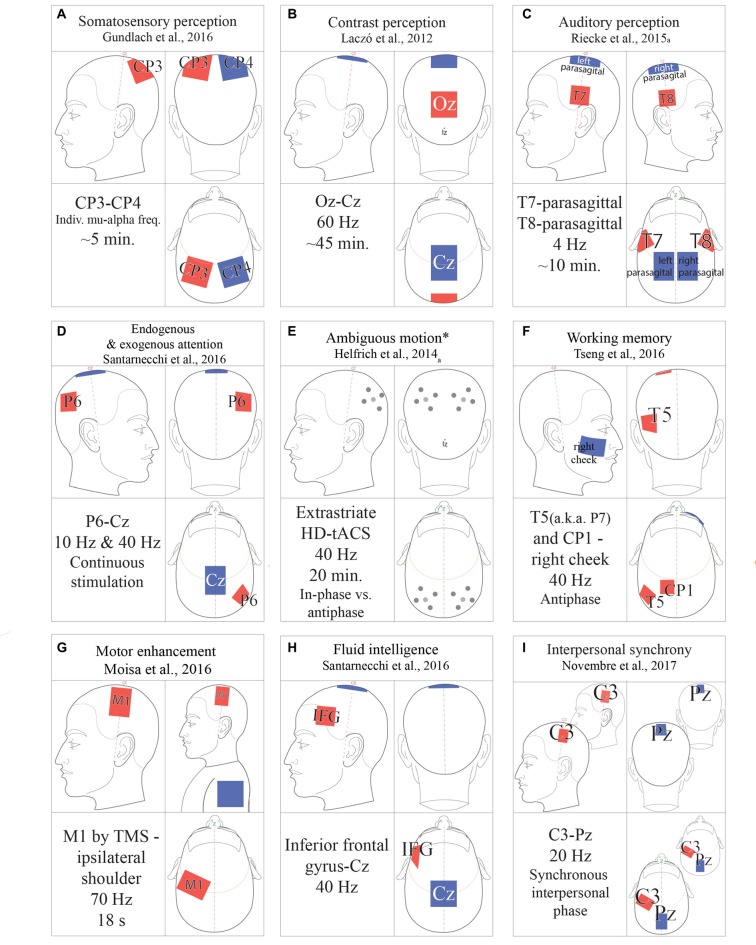
tACS protocols. **(A)** 1000 μA; **(B)** 1500 μA; **(C)** >1000 μA; **(D)** 2000 μA; **(E)** Amplitude thresholded; **(F)** 1500 μA; **(G)** 1000 μA; **(H)** 750 μA; **(I)** 1000 μA. All amplitudes in peak-to-peak microamps. *Ambiguous motion protocol alternates both phase-offset and HD-montage.

Despite a number of tACS studies (Figures 1A, 2A–I), consensus has not yet been reached on tACS methods. A methodological gold-standard would ensure reliable results and systematic improvement of the field. Several rigorous tACS methods have been proposed, such as double-blind and active sham conditions (Brignani et al., 2013; Gall et al., 2016). This mini review examines several articles that applied strict experimental controls and proposes an optimized tACS protocol for future studies. It is intended to highlight: (1) key advantages of tACS; (2) theoretical and practical assumptions about tACS; and (3) proposal of tACS protocols. Please refer to the following articles for a more comprehensive review (Veniero et al., 2015; Schutter and Wischnewski, 2016; Matsumoto and Ugawa, 2017).

## The Advantages of tACS: The Causality

### Transcranial Current Stimulation Waveforms

Recent interest in neuromodulation lies partly in the desire to investigate cognitive function in a parametrically rigorous manner. Differences in current profiles distinguish the forms of tES. In tACS, the oscillating current rhythmically reverses the electron flow. Unlike transcranial direct current stimulation (tDCS), averaging over a full cycle, the tACS current omits the directional voltage component (Figure 1B). Other methods include oscillating tDCS (otDCS), where oscillations are oriented by a direct component (Guleyupoglu et al., 2013), and transcranial random noise stimulation (tRNS) which injects an alternating current of bounded stochasticity (Saiote et al., 2013). Although different stimulation protocols have not fully revealed the neurophysiological mechanisms of each method, we now conclude that the oscillatory state predicts cognitive phenomena (Wang, 2010; Donner and Siegel, 2011; Schutter and Wischnewski, 2016).

The advantage of tACS, unlike other types of NIBS, is that it enables manipulation and entrainment of intrinsic oscillations through the injection of sinusoidal currents (Paulus, 2011; Thut et al., 2011; Antal and Paulus, 2013). The phase profile of the tACS current alternates regularly between positive and negative voltages. By contrast, with tDCS, the current describes a monophasic, distinctly non-oscillating baseline voltage. Endogenous activity is modulated by depolarization (anode) or hyperpolarization (cathode) in the global flow of current, which supplies electrons to the anodal electrode (promoting endogenous oscillations) and retracts electrons from the cathodal electrode (suppressing endogenous oscillations; Song et al., 2014).

### tACS Entrainment and Neuroplasticity

The most prominent mode of neuroplasticity, long-term potentiation (LTP; Lee and Silva, 2009), is manifested by spike-time dependent neurophysiology. Through LTP, the suprathreshold spike activity of neurons enhances the neuronal connection and signal propagation through post-synaptic dendrites. This suggests that frequency and phase information are fundamental parameters of neural function (Herrmann et al., 2016). Unlike tDCS, an advantage of tACS is that it permits physiological entrainment through frequency stimulation at nearly imperceptible current strengths. Endogenous oscillations during entrainment are synchronized with extrinsic, rhythmic stimuli (Herrmann et al., 2013). Researchers have effectively used rhythmic photic stimuli (Adcock and Panayiotopoulos, 2012; Poleon and Szaflarski, 2017) to analyze the visual cortex for sensitivity to entrainment (Adrian and Matthews, 1934). An advantage of tACS is that it can completely bypass sensory stimuli (Figures 2A,B,C,E) by inducing entrainment through externally applied and almost imperceptible alternating currents. To clarify the causal relationship between cognitive function and oscillatory activity, the combination of behavioral (Kanai et al., 2008; Feurra et al., 2011a,b; Laczó et al., 2012) and electrophysiological (Zaehle et al., 2010) methods are required (Thut et al., 2011; Herrmann et al., 2016). This joint approach should be performed based on clear physiological assumptions.

tACS can parametrically control neurophysiology on the assumption that endogenous oscillation is constituted by interactions with oscillatory inputs from near and eccentric neural sources (Herrmann et al., 2016; Romei et al., 2016; Vosskuhl et al., 2016). Fourier transformations of these aggregate neural oscillations can be recorded and computed by EEGs, and show functional bands of activity that are sensitive to entrainment (Thut et al., 2011; Herrmann et al., 2016; Romei et al., 2016). Entrainment with tACS therefore offers a functional advantage. Multiple sessions of tACS entrainment showed a significant improvement in implicit motor learning (Antal et al., 2008). Neuroplastic changes were also found in the presence of tACS aftereffects (Vossen et al., 2015). There remains an open question as to whether our neurology is predisposed to replicate the natural frequency of the environment (entrainment) or whether the exogenous frequency changes the physical connectivity through spike time dependent plasticity (Zaehle et al., 2010; Vossen et al., 2015; Kasten et al., 2016).

### Causal Inferences Using tACS

Electrophysiological measurements are dependent variables and cognitive processes are independent variables (Herrmann et al., 2016). Unlike conventional electrophysiological measurements, NIBS allows a deeper understanding of cognitive processes by reversing the traditional dependence of variables (Poldrack, 2006). EEG studies deal with many terrains in the oscillatory phenomenology of cognition and behavior. Well-defined cognitive functions are commonly attributed to certain oscillatory features and frequencies. Based on such established electrophysiological evidence, researchers use tACS to extend the causal explanation of electrophysiological variables into cognitive processes. Thus, an advantage of tACS is in measurement of behavior as a function of parameterized electrophysiological manipulation.

### Recent Focus on Determining Behavioral Causality from Neurophysiology

For causal interpretation of neural systems and functional circuits, neuromodulation is required at multiple scales of cortical network activity (Ruffini et al., 2014). Although visualization of voltage-related neurophysiology using multi-photon microscopy is still nascent, it has been shown that large-scale neurophysiological dynamics in animal models can be attributed to optogenetics and invasive electrophysiology (Ali et al., 2013; Kuki et al., 2013; Anastassiou and Koch, 2015). To investigate functional circuits, clinicians derive functional connectivity from the combination of intracranial neuromodulation with tractography (Elias et al., 2012). Invasive investigations in human electrophysiology, however, have only achieved local entrainment using alternating currents (Amengual et al., 2017). The effect of a 50 Hz current on memory performance has been shown by deep brain stimulation of the human hippocampus (Ezzyat et al., 2017). Non-invasive stimulation methods have been shown to result in neuroplasticity in multiple functional areas (Bolognini et al., 2009; Hameed et al., 2017). tACS has been shown to be more effective than tDCS for network entrainment (Ali et al., 2013), and closed-loop tACS in epileptic animal models has been shown to alleviate the spike-and-wave effects that are noticeable during seizures (Berényi et al., 2012). Thus, research into neural systems reveals the functional role of neural oscillations in a wide range of scales, models and contexts.

### Feedback-Controlled EEG-tACS for Stronger Inferences

Feedback-control enables precise modulation of endogenous oscillations. Another advantage of tACS is that it can be combined with other non-invasive neuromodulation techniques and EEG to enhance experimental inference (Kanai et al., 2010; Boyle and Fröhlich, 2013; Roh et al., 2014; Lustenberger et al., 2016; Raco et al., 2016). The rigorous experimental setup of simultaneous stimulation and electrophysiology requires the ability to address stimulation artifacts. Although many EEG-tACS experiments have recorded pre- and post-stimulus EEGs to avoid signal artifacts (Zaehle et al., 2010; Veniero et al., 2015), recent improvements in simultaneous EEG-tACS better elucidate endogenous oscillations (Roh et al., 2014; Dmochowski et al., 2017; Neuling et al., 2017). Although EEG-tACS enables temporal resolution of less than a millisecond (Neuling et al., 2015; Ten Oever et al., 2016), concerns about signal artifacts persist (Noury et al., 2016). The closed-loop tACS-TMS protocol may also parameterize the magnetic stimulation to instantaneous physiology (Thut et al., 2017). For example, Raco et al. (2016) developed a closed-loop protocol that uses instantaneous tACS phase-triggered TMS pulses. Some research questions do not require an online protocol, but future combinations of these techniques can help to understand the causal relationship between brain oscillations and behavior (Herrmann et al., 2016; Lustenberger et al., 2016).

## Methodological Considerations for Rigorous tACS Experiments

The tACS methodology has not yet been standardized. In order to obtain valid results, all modes of stimulation must be performed while taking precautions to increase replicability and validity. Multiple sessions of tACS can be effective (Antal et al., 2008). Optimized effect sizes can be achieved in part through rigorous testing of neurologically parsimonious frequency and amplitude ranges. With regard to effect-size, Ali et al. (2013) built a computational model of tACS stimulation in anesthetized ferrets to investigate the effect of tACS on large-scale cortical network activity. They found that tACS had a greater impact on network resonance than tDCS (Ali et al., 2013).

Subject-awareness of condition assignments should be avoided through established blinding methods. Although tACS does not emit an audible signal of stimulus amplitude and frequency, as with TMS, blinding should be enforced by applying a subject-specific stimulus detection threshold for both visual and somatic perception. The rostral electrode montage is known to more easily induce phosphenes (Neuling et al., 2012; Schutter, 2016)—the perception of light that is purely neural and non-photic in origin. With active-sham control, the experiment can be tightly controlled by changing only the montage or frequency while limiting all other parameters (Mehta et al., 2015; Guerra et al., 2016; Schutter, 2016). Both sham and experimental currents should incorporate the same ramping period so that the participants cannot distinguish the condition (Woods et al., 2016). Future research can further promote replicability by implementing double-blinding through an automated tACS protocol wherein both subject and experimenter are naïve to condition assignments.

Given the neuro-oscillatory effects of sensory input, the environment is particularly important in tACS experiments where the endogenous state of the subject is the focus of investigation (Reato et al., 2013). The lighting conditions can influence the detection threshold for tACS-induced phosphenes (Kanai et al., 2008; Paulus, 2011; Neuling et al., 2013). Some researchers suggest that the tACS electric field is lower than the threshold of retinal sensitivity, but others suggest that dark adaptation of the retina contributes to the frequency at which retinal excitation occurs (Kanai et al., 2008; Paulus, 2011; Herrmann et al., 2013; Neuling et al., 2013). Another advantage of tACS is the modulation of the individual alpha frequency (IAF). In another vein, eyes-closed EEG states are represented by high baseline alpha-band power (Herrmann et al., 2016). Predictably, Neuling et al. (2013) used within-band tACS to increase IAF power, specific to eyes-open conditions. Reproducible tACS results thus require investigators to treat neuromodulation as a function of the contextual brain state predictor.

While tACS protocols vary significantly, general refinements, optimal protocols, and function-specific parameters have emerged (Fröhlich, 2016). To avoid visual artifacts while targeting rostral cortical regions, investigators can improve the localization of the current with “ring” electrode montages. Here, a single stimulation electrode is encircled by four reference electrodes (Figure 2E; Helfrich et al., 2014a). Stimulation frequency is also subject to tailoring. Many experiments predetermine the stimulation frequency for all subjects (Moisa et al., 2016; Riecke, 2016). Between-subject variability in EEG band power, such as 7–12 Hz alpha, has led researchers to personalize the stimulation frequency, determined by peak band power (Mehta et al., 2015; Herrmann et al., 2016). The reference electrode is crucial to achieve the desired current density (Figure 1C) and stimulus. Mehta et al. (2015) compared the peak physiological tremor associated with the contralateral reference electrode as well as the extracephalic electrode placed on the ipsilateral or contralateral shoulder. They determined that only the contralateral extracephalic reference montage entrained the peak physiological tremor (Mehta et al., 2015). Additionally, multi-electrode montages can be applied to multiple electrical currents, either in-phase or out-of-phase, to investigate inter-hemispheric coherence (Helfrich et al., 2014b; Strüber et al., 2014).

Another methodological dimension is the subject experience. The advantage of tACS over TMS is that tACS omits high amplitude magnetic pulses and exhibits fewer reported side effects such as muscle twitching, discomfort and nausea (Rossi et al., 2009). Given individual variability in autonomic arousal (Wenger et al., 1961), before initiating an experiment, experimenters should resolve subjects’ anxieties about the electrical current through brief current exposure. This humane precaution may reduce artifacts in both oscillatory and behavioral data (Bonnet and Arand, 1997).

Finally, researchers generally assume the safety of tACS within the guidelines, but continuous improvement of subject safety and tACS methods requires constant monitoring of potential behavioral changes that can persist beyond post-stimulation measurements. Parameter-specific investigations of these aftereffects continue to emerge (Antal et al., 2008; Neuling et al., 2013; Wach et al., 2013; Veniero et al., 2015; Herrmann et al., 2016; Matsumoto and Ugawa, 2017).

## Target and Task-Specific tACS

### Attention

tACS has been used in direct investigations of endogenous and exogenous attention (Figure 2D). Hopfinger et al. (2016) investigated the effects of alpha and gamma tACS on endogenous and exogenous attention by comparing subjects’ performance on two spatial cueing tasks. In their study, 40 Hz gamma tACS facilitated endogenous attention, but had no significant effect on exogenous attention, suggesting a critical role of low gamma in attentional disengagement and reorientation (Hopfinger et al., 2016).

### Perception

Using EEG, researchers pursued the oscillatory correlates of perceptual phenomena such as the ventriloquism effect (Kumagai and Mizuhara, 2016), the double-flash illusion (Cecere et al., 2015) and mirrored social embodiment (Oberman et al., 2005; Raymaekers et al., 2009). Leveraged by common electrophysiology, tACS has proven useful for perception studies.

There is growing evidence of the consequences of tACS on audition (Figure 2C; Baltus and Herrmann, 2016; Riecke, 2016). Neuling et al. (2013) found a causal relationship between oscillatory phase and auditory signal detection by applying a 1000 μA DC current, summed with an approximately 425 μA, 10 Hz component. Although this study was not a pure instantiation of tACS, other studies of oscillation and audition have demonstrated the functional role of alpha (Weisz et al., 2011), and the delta and theta frequencies (Riecke et al., 2015a,b). Speech perception can also be modulated using 40 Hz tACS (Rufener et al., 2016a,b).

Protocols specifically targeting the visual cortex face significant difficulties despite the innovation of the tACS montage. For example, some have suggested the active stimulation to Oz and the reference stimulation to Vertex (Cz), but purely non-retinal stimulation of the visual cortex remains somewhat controversial (Antal and Paulus, 2013; Schutter, 2016). Other studies applied montages anterior to the occipital cortex, and the induction of retinal phosphenes did not emerge as a significant confound of experimental results (Kirov et al., 2009; Pogosyan et al., 2009; Kanai et al., 2010). Rigorously controlled tACS currents allow an array of neuroscientific investigations.

In the visual domain, investigators have successfully modulated motion perception (Helfrich et al., 2014a, 2016; Strüber et al., 2014), mental rotation (Kasten and Herrmann, 2017), visuo-motor coordination (Santarnecchi et al., 2017), and induced phosphenes (Kanai et al., 2008). The tACS literature pays significant attention to controlling the phosphene and allowing subjects to experience an experimentally useful phosphene in a parameterized manner (Schutter, 2016). Care should be taken to determine subject-specific parameters for phosphene-induced thresholds and to perform experiments at or below these thresholds (Kanai et al., 2008). Many researchers also asked subjects about phosphene perception (Antal et al., 2008; Strüber et al., 2014; Schutter, 2016). Investigators of tACS-induced phosphenes compared current profiles in light and dark conditions (Schutter, 2016). Kanai et al. (2008) induced qualitative changes in phosphene perception, such as differences in position, orientation, diffusivity and temporal stability (flickering). In lighted conditions, stimulation in the beta range (20 Hz) resulted in low phosphene detection threshold and qualitatively strong phosphenes, whereas in the dark condition stimulation in the alpha range (10–12 Hz) induced the strongest phosphenes. Thus, the frequency range from 10 Hz to 40 Hz (Moliadze et al., 2010; Paulus, 2011) has an effect on phosphene interference. Beyond phosphenes, Strüber et al. (2014) demonstrated that the perceived direction of apparent motion can be modulated in an ambiguous motion task by applying bilateral, anti-phase tACS in the gamma band (Figure 2E).

### Motor Function

tACS has been used to investigate motor enhancement (Figure 2G), learning and memory. While appropriate montages at C3/C4 in the international 10-20 system target the contralateral limb, motor regions of interest exist outside the motor cortex. A tACS-fMRI investigation revealed that the behavioral change was positively correlated with BOLD activity in primary motor cortex (M1) but it was negatively correlated with activity in dorsomedial prefrontal cortex, a region regarded as a locus of executive motor control (Moisa et al., 2016). Brinkman et al. (2016) compared alpha and beta tACS to investigate movement selections. Enhanced movement acceleration and velocity were achieved with gamma band entrainment of the M1 (Moisa et al., 2016), and sensorimotor integration was promoted by beta band entrainment (Guerra et al., 2016). A previous study showed that alpha-band tACS decreased corticomuscular coherence over 30 min after stimulation (Wach et al., 2013). Motor learning was improved by applying 10 Hz tACS (Nitsche et al., 2003; Antal et al., 2008), and motor memory was enhanced by applying tACS during sleep (Lustenberger et al., 2016).

Following on animal models of cerebellar physiology (Ohyama et al., 2003; Ohmae and Medina, 2015; Giovannucci et al., 2017), there is a nascent implementation of cerebellar tACS in investigations of human motor function (Tremblay et al., 2016). Importantly, cerebellar neurophysiology excludes recurrent excitatory loops (Buzsáki, 2006; Rokni et al., 2008, 2009; Duguid et al., 2015). Still, recent studies showed that cerebellar tACS can lead to improved cortical excitability and motor behavior (Naro et al., 2016, 2017). Moreover, non-invasive neuromodulation of the spine improved locomotor function in both paralyzed and non-injured individuals (Gerasimenko Y. et al., 2015; Gerasimenko Y. P. et al., 2015).

### Memory, Learning and Higher Cognition

tACS has been used to investigate memory, learning and higher cognitive function. Notably, an investigation of forebrain functions, such as working memory (Figure 2F), should consider visual artifacts while using rostral montages. For visual memory-matching tasks, a previous study compared the performance between in-phase, bilateral, theta-band stimulation and anti-phase stimulation (Polania et al., 2012). In-phase theta has been shown to reduce reaction time in the visual memory-matching task, whereas anti-phase has been shown to degrade performance and increase reaction time (Polania et al., 2012). Alekseichuk et al. (2016) found that spatial working memory depends on theta-gamma, cross-frequency coupling. An experiment applying feedback-controlled 12 Hz tACS stimulation during sleep showed no significant increase in declarative memory consolidation, despite increased motor memory consolidation and sleep spindle activity (Lustenberger et al., 2016). In addition, participants who were stimulated with theta (6 Hz) over the frontal cortex experienced faster reversal learning (Wischnewski et al., 2016).

Interhemispheric phase-difference appears to influence executive decision-making. An investigation of risk-taking using the Balloon Analog Risk Task found that theta (6.5 Hz) stimulation of the left hemisphere can increase risk-taking behavior (Sela et al., 2012). A bi-frontal, anti-phase protocol used in the above investigation of reversal learning by Wischnewski et al. (2016) should also be noted for an unexpected increase in risk-taking behavior.

## Summary and Future Directions

Our goal was to clarify the benefits of tACS on neural investigations. Despite many methodological advances, unraveling the neurophysiological and oscillatory complexity of cognitive function requires investigation at multiple scales from cells, to animal neurophysiology, and finally, to clinical trials.

Investigators studying cognition through psychophysical measures suggest a vital role played by system-level information integration, such as audio-visual integration (Shams et al., 2000; Bhattacharya et al., 2002). While deep-brain integrators of neuronal information have been identified in animal models (Fetsch et al., 2013), causal determinations have been significantly more difficult in humans (Beauchamp et al., 2004). However, the electrophysiological literature suggests the emergence of perception, cognition and consciousness from the integration of endogenous, oscillatory information. The manipulation of large scale brain oscillations by tACS may provide further insight in this area.

Neuromodulation has widespread potential in investigations of the functional role of oscillatory neural information. The role of phase and frequency information has already been documented in neuropathologies such as schizophrenia (Perez et al., 2017), epilepsy (Chu et al., 2017; Nariai et al., 2017), and Parkinson’s disease (Brittain et al., 2013; Cozac et al., 2016; Latreille et al., 2016). Given the difference in EEG oscillations between coma, sleep and awake states, whether tACS can control perioperative EEG to reduce the dose of anesthesia, maintain unconsciousness during operation, and even promote recovery from anesthesia is an open question. The industry can expect applications to the brain-computer interface as well as online modulation of cognitive states for human operators. Attention control also has wide-spread industrial applications by allowing participants to easily integrate environmental cues and information.

Here we have introduced strictly controlled tACS protocols, methodological precautions, and guidelines for future tACS implementation. Recent developments in tACS scholarship, which are only partially presented, signal the intersection of numerous neurocognitive specializations, focused on the central role of oscillatory activity in brain function, cognition and behavior.

## Author Contributions

AVT and KY compiled literature for this review. KY provided mentorship on methods, theory and prose. AVT wrote the manuscript and provided illustrations.

## Conflict of Interest Statement

The authors declare that the research was conducted in the absence of any commercial or financial relationships that could be construed as a potential conflict of interest.
